# Assessing the asymmetric effects of capital and money markets on economic growth in China

**DOI:** 10.1016/j.heliyon.2022.e08794

**Published:** 2022-01-21

**Authors:** Mohammad Naim Azimi

**Affiliations:** Faculty of Economics, Kabul University, Afghanistan

**Keywords:** ARDL, Symmetries, Asymmetries, Money market, Capital market

## Abstract

This study examines the effects of capital and money market predictors on economic growth in China using non-linear autoregressive distributed lags and dynamic multiplier methods. Applying asymmetric techniques is based on the hypothesized linear effects of finance on growth. Confirming the asymmetric nexus and long-run bounds amid indicators, the results demonstrate that positive (negative) shocks from money market rate decrease (increase) economic growth, while negative (positive) shocks from real interest rate and total liquidity increase (decrease) growth in the short-run. Besides, the results reveal that the shocks (positive and negative) from market capitalization and stock market turnover increase economic growth, while the shocks from total stock traded decrease growth both in the short and long runs. Moreover, the results of error-correction reveal a steady speed of adjustment of the short-run asymmetries to their long-run equilibrium, implying that improved financial systems attract sound financial projects, leading to sustainable and long-run economic growth. In light of the findings, relevant policy recommendations are discussed.

## 1 Introduction

The contributions made by capital and money markets have proven their important nexus with economic growth and are well recognized as significant intermediate conduits driving economic growth by scholars (Bukowski and Zięba, 2019; Destek et al., 2020; Haseeb et al., 2019; Hassan et al., 2020; Karimo and Ogbonna, 2017; Le, 2020; Le et al., 2019; Lestari et al., 2020; Ncanywa and Mabusela, 2019; Twinoburyo and Odhiambo, 2018), academics, and policymakers. But it is important to understand the magnitude of the effects of capital and money market indicators on economic growth, which is truly essential to help policymakers construct specific policies in light of the macroeconomic variables (Chen et al., 2020). Though there are many empirical works about the nexus between economic growth and financial intermediaries in the U.S., western regions (Jula and Jula, 2013; Burhop, 2006; Levine et al., 2000; Bass, 2018; Purewal and Haini, 2021; Swamy and Dharani, 2020; Coccorese and Silipo, 2015; Shobande and Ogbeifun, 2021), and some recent studies for China that have postulated impenetrable results assuming symmetrical nexus amid finance and growth (Burzynska, 2009; Yaoling, 2009; Wang et al., 2010; Ouyang and Li, 2018; Wu et al., 2020; Guo and He, 2020), yet studies are scarce for China. Nevertheless, considering the existing literature on the symmetrical nexus between finance and growth and the confounding results presented by recent studies, there is still an obvious gap in the literature, which is filled by the present study. In particular, most of the recent empirical studies in China have assumed that the relationship between finance and growth is symmetrical and they have used linear methods such as vector error-correction, linear autoregressive distributed lags, vector autoregressive, and linear cointegrating regressions (see, for example, Aziz and Duenwald, 2002; Peng, 2019; Maswana, 2009). In contrast with the existing literature for China, the present study develops three competing hypotheses. H_1_: the finance-growth nexus is asymmetric in both the short and long run; H_2_: the money market, capital market, and economic growth all have long-run bounds; and H_3_: both money market and capital market indicators have an asymmetric effect on economic growth. To test the hypotheses, this study employs the non-linear autoregressive distributed lags model which is augmented with well-known capital and money market variables and the dynamic multipliers method to assess the response of economic growth towards the shocks released by capital and money market predictors.

Back to the growth puzzles, it is important to explore the contribution of an underdeveloped financial system to a rapidly growing economy in China. Although China has witnessed substantial reforms in the past four decades, the structural improvement in the financial sector of the country remains lagging. Before 1949, the financial system of China was well-developed, witnessing a swift growth in the number of privately owned banks. But since the conversion of capitalist firms into nationalized ones with the establishment of the “Peoples' Republic of China”, there has only been the People's Bank of China, possessed and managed by the ministry of finance, owing more than 90% of the financial assets, governing the system, and dealing with almost all the financial transactions in China (Allen et al., 2017). Despite noticeable centralization, more banking and nonbanking financial intermediaries emerged during the 1980s. For most of the 1990s, two stock markets were established. (i) the Shanghai Stock Exchange and (ii) the Shenzhen Stock Exchange, both of which expanded during the 1990s and 2000s. In the same span of time, the real estate market has also emerged which has gained comparable size during the year 2007. In 2020, the total market capitalization of China touched its peak ever and exceeded almost 79 trillion yuan, constituting about 59% of its nominal GDP. China's stock market is one of the world's most short-term speculative and highly volatile due to a large number of investors.

According to Huang and Wang (2017) China has built a wide-ranging financial system, but it has not permitted the open market mechanism to operate freely as it is based on the dual-track reform strategy of the country. The foretold strategy imposed between the state and non-stated sectors did not cause the economy to suppress; rather, it caused greater financial risks such as the temporal fluctuations of firms' contributions to the systematic risk, financial institutions’ sensitivity to the systematic risk, and the long-run risk of capital shortfalls (Zhou et al., 2020; Derbali, 2017). This in turn affected economic efficiency, because it heavily relied on capital investment and credit as the key drivers of economic growth. This reliance has posed significant vulnerability and risk to the financial system of China (Dieppe et al., 2018; Jiang et al., 2020). Recognizing the existing risks to the financial system, it is further important to support the unconventional financial sectors that play a significant role in stimulating economic growth. According to Allen et al. (2005) it is not only the formal financial sectors that spur economic growth; rather, one can symbolize the role of the informal financial sectors as an alternative driver of growth that supports all dynamic sectors of the economy.

Pan and Mishra (2018) state that China's rapidly growing stock market does not have a significant impact on the real economy in the short run; rather, state-owned monopolies have a greater impact on China's economic performance because they encourage economic growth in the short run. Though the linearity assumption in testing the effects of the financial market is critical, Ouyang and Li (2018) document the negative impact of money supply, credit to the insurance industry, and stock market value on economic growth in China. One can note that the stock market has had rapid growth in China since its re-establishment, but is still categorized as an emerging market compared with the developed markets. This is because China's stock markets are heavily dominated by naive retail investors (Li, 2017), while the developed markets are dominated by stylized institutional investors (Ding and Zhong, 2020). In recognition of institutional investors' role in developing further efficient capital markets, China has imposed a number of policy measures to promote the role of institutional investors in contributing to economic growth.

Nonetheless, considering the participants in China's stock markets, another key factor that keeps China's stock markets apart from the world's developed stock markets is the short-selling practice. Practically, short-selling is regularly practiced in the developed markets, which was not allowed in China till the middle of 2010, and is highly controversial as to whether it impacts the capital markets (Chen et al., 2020). Empirically, short-selling mends the price efficiency through the asymmetric channels of information and liquidity, whereas margin trading improves the efficiency through the channels of ownership scale.

The present article is intensely different from recent studies and contributes to the existing literature in three ways. First, using the principal component analysis method, this study perceives composite indicators with respect to the capital and money markets that asymmetrically affect economic growth. Second, the author employs the asymmetric ARDL model to permit the capital and money market variables to implore the positive partial sum of squares, the negative partial sum of squares, and their effects on economic growth by controlling for the basic macroeconomic variables like net foreign direct investments and capital investments. Third, the author investigates asymmetric shocks to economic growth as the response of the growth to the newly established dynamic equilibrium from the previous period's dynamic disequilibrium using the dynamic multipliers of capital and money market indicators. The most interesting results noted by the present study are also threefold. First, it is evident that the finance-growth nexus is asymmetrical. Second, all capital and money market variables asymmetrically impact economic growth in the short run, while capital market indicators affect growth both in the short and long runs. Third, it is also evident that economic growth swiftly responds to the plummets in the capital and money markets. The remainder of this article is organized as follows: The section “Literature review” reviews the theoretical foundation of the growth-finance nexus and discusses the relevant empirical literature. The section “Methods” discusses the data and variables of the capital and money markets and the econometric techniques used to analyze them. The section “Results and discussions” presents the results of the study and discusses the analysis. The section “Conclusions” concludes the article and offers a set of policy measures based on the findings that suit the economic growth in China.

## 2 Literature review

The literature vastly documents that efficient financial systems ease capital accumulation and mobilization to boost sufficient liquidity and credit availability to spur economic growth. Therefore, an improved financial system to affect economic growth is based on five conduits: (i) improved human resource management; (ii) boosted capital accumulation and its efficient allocation; (iii) savings mobilization; (iv) interest rate liberalization; and (v) financial risk management and mitigation (Levine, 1997), implying that boosted capital accumulation facilitates higher savings and investments that improve the economic scale and materialize real economic endeavors (Nyasha and Odhiambo, 2017). Besides that, an improved financial system reduces the cost of transactions, controls the skewness of financial information, reduces risks, and makes available capital accumulation and allocation both for the public and private sectors to contribute to economic growth (King and Robson, 1992; Chakravarty, 1964; Massell, 1962). Therefore, an improved financial system is an essential factor that drives economic growth. The initial modern thinking on the theoretical framework of the finance-growth nexus is traced back to the study conducted by Bagehot in the 1870s, which demonstrated how financial systems are connected with economic growth (Stolbov, 2013). This prediction was based on standard neoclassical demand and supply theory, which was supplemented by arbitrage theory (Marwa and Zhanje, 2015).

On the one hand, the existing literature reports a large number of studies and documents the significant contribution of the financial system to economic growth, though the reported contributions are still unconvincing and arguable; while on the other hand, studies analyzing the asymmetric effects of the financial system on economic growth are quite exceptional and rare in various economic contexts. Even if they did, they have nonetheless postulated baffling results, putting the asymmetries of real economic growth in auxiliary debate or denying the critical requirement for knowledge of the asymmetric effects of macroeconomic indicators. Regardless of the paucity of the non-linear nexus between the financial system and economic growth and the specific economic context of lower or middle class economics and the rest of the economic classes, the existing literature contains few empirical studies that attempted to investigate the financial system and the economic growth nexus, in which Schumpeter (1934) and Patrick (1966, 1972) were the first authors who posited the nexus of the financial system with economic growth based on (i) the demand-led assumption, (ii) the supply-led assumption, and (iii) the development stage assumption.

Whereas (i) hypothesizes the dynamic economic growth that spurs and entices the financial system and is tense to the distinctive response of the financial system, further structural improvements ease the productivity and enhance the required financial development that errands the financial systems to suit economic growth (Manta et al., 2020; Odhiambo, 2004, 2008; Rachdi, 2011), while (ii) assumes that the financial system spurs economic growth and infers that suitably deliberated financial market reforms upkeep the efficiency with which capital accumulation and investments boost economic growth (see, for instance, Levine, 2003; Calderón and Liu, 2003; Demirhan et al., 2011; Kar et al., 2011), and (iii) assumes that the consideration of the development stage describes a development transference between (i) and (ii) hypotheses that finally cause economic growth and improve the financial system. That is, originally, the appropriate integration of economic growth, which boosts production and offers the essential arrangements that improve the integration of the financial system. This way, higher foreign capital, technologies and skills flow through the liberalization and enhance their leverage to economic growth as the supply-led improves in the long run (Patrick, 1966).

Considering these intuitions, recent studies have posited various findings with respect to the financial system's effects on economic growth. Therefore, the present study explores these studies and pledges a one-way causality that can either be growth-led or financial system-led. Abu-Bader and Abu-Qarn (2008) explored the causal nexus between economic growth and financial development for six Middle Eastern and North African countries, including Algeria, Egypt, Israel, Morocco, Syria, and Tunisia. The authors employed the vector autoregressive of Toda-Yamamoto and reported strong statistical evidence to support the hypothesis that finance leads to growth in Algeria, Egypt, Morocco, Syria, and Tunisia, but weak causality for growth-finance in Israel. Masih et al. (2009) tested the demand-led and supply-led hypotheses for Saudi Arabia and used the error-correction and variance decomposition methods. The author found evidence supporting the supply-led and rejected the demand-led hypotheses. Augustine and Pius (2010) examined the impact of stock market development on economic growth in Nigeria using the ordinary least square and found that the stock market size and the turnover ratios are significantly positive to explain the economic growth, but the stock market liquidity is negative to affect the growth in the long-run.

Kar et al. (2011) investigated the direction of causality between financial development and economic growth in the Middle East and North African countries using the Seemingly Unrelated Regressions and Wald tests with the country-specific bootstrap critical values. The authors found that there is no significant harmony with respect to the causality direction between finance and growth in light of the country-specific observation. Bojanic (2012) explored the growth-finance-trade nexus and employed the Granger regression and error-correcting models on a set of time series data for Bolivia. The authors provided statistical support for a long-run relationship between economic growth, financial development, and trade openness, while they also noted that there is only a unidirectional causality from financial development and trade openness to economic growth.

Shahbaz et al. (2013) tested the economic growth nexus with energy consumption, financial development, trade openness, and CO_2_ emissions for Indonesia using a quarterly dataset from 1975Q1-2011Q4 and employed the autoregressive distributed lags bound testing method to ascertain the long-run memory between the indicators. The authors found that the indicators are cointegrated and the causality supports the feedback hypothesis between the indicators, while their results also open up new policy insights to consider financial development and trade openness as key factors that stimulate environmental quality. In exploring the empirical literature, Alkhuzaim (2014) employed cointegration analysis and Granger causality techniques based on error correction on a set of data from 1992 – 2012 for Qatar. The author found that there is a positive long-run nexus between financial development and economic growth, and that there is a bidirectional causality between broad money and economic growth as well as a unidirectional causality between domestic credit and economic growth.

Unlike the existing studies for Nigeria that symmetrically tested the finance-growth nexus, Adeniyi et al. (2015) re-examined the relationship between financial development and economic growth by applying asymmetric regression to a set of time-series data from 1960–2010. The authors provided statistical evidence that financial development has a negative impact on economic growth but a sign reversal results in considering the threshold-type effects as using a composite index of FD led to a similar outcome for Nigeria. Moreover, Contuk & Güngör (2016) investigated the finance-growth relationship using quarterly data from 1998 to 2014. Employing Granger causality techniques, their results reveal statistical support for both the supply-led and the demand-led hypotheses. Similarly, Nyasha & Odhiambo (2017) examined the effect of both bank- and market-based financial development on economic growth using a set of data from 1980 to 2012 for Kenya. The authors used the autoregressive distributed lag bounds testing approach and found that market-based financial development positively affects economic growth and that bank-based financial development has an insignificant impact on economic growth.

Further review of the existing literature reveals that after the global financial crisis in 2008, some posit a positive nexus between financial liberalization and economic growth, while others posit a negative nexus between financial liberalization and economic growth. With these insights, Batuo et al. (2018) examined the relationship between financial instability, financial liberalization, financial development, and economic growth for 41 African economies using a set of data from 1985–2010. The authors found that financial development and financial liberalization positively affect financial instability, while economic growth minimizes financial instability. Pinshi (2020) examined the direction of causality between financial development and economic growth in the Democratic Republic of the Congo using a set of data from 2004 to 2019. The author found that the long-run is insignificant, but there is a short-run causality between the indicators, concluding that financial development leads to economic growth.

Nguyen et al. (2021) investigated the influence of economic growth, financial development, transportation capacity, and environmental degradation using a symmetric autoregressive distributed lags model on a set of data from 1986 to 2019. The authors showed that there is a significant long-run memory among the variables, and their results provided statistical evidence that an increase in economic growth and financial development deteriorates the environmental quality, while transportation capacity and foreign investment provide a reversible feedback. As a general approach to testing the finance–growth nexus, almost all of the recent studies assumed linearity and employed symmetric techniques such as cointegrating regressions, vector autoregressive, vector error-correction, autoregressive distributed lags, Granger causality, and ordinary least squares, whereas a few studies assumed non-linear relationships between the financial system and economic growth and applied asymmetric techniques. These authors are Ehigiamusoe and Narayanan (2019), Tsagkanos et al. (2019), and Shodipe and Shobande (2021), who studied, respectively, in a wide-ranging economic context, Greece, and the U.S., but there is yet no study concerning the asymmetric effects of the financial system on economic growth in China, which is the fastest-growing economy in the world. This article is a distinctive work that significantly contributes to the literature and fills the existing literature gap that has been missed by recent studies (see Table 1 for a summary of some relevant empirical studies with regards to China's economy).

**Table 1:** A summary of some relevant empirical literature.

Author(s)	Economy	Dataset	Method	Assumption	Findings
Aziz and Duenwald (2002)	China	1988–1997	Panel regression	Symmetric	In addition to the existence of a nexus between financial markets and economic growth, domestic investments play a relatively small role in economic growth compared to foreign investment.
Shan and Jianhong (2006)	China	1978–2001	VAR, IRF	Symmetric	Financial development affects economic growth as the second force after labor inputs in China. Financial development and economic growth show bidirectional causality and thus support the finance-led growth hypothesis.
Maswana (2009)	China		Granger causality	Symmetric	The author posits that there is a bidirectional causality between economic growth and the financial development indicators.
Peng et al. (2014)	China	1978–2004	Delphi, PCA, VAR	Symmetric	The authors find evidence of significant positive effects of liberalization on growth in the short run and on accumulated growth in the long run.
Kandil et al. (2017)	China and India	1970–2013	Panel cointegration	Symmetric	The authors find that in the long run, financial development improves economic growth both in India and China, but globalization accelerates economic growth only in India.
Pan and Mishra (2018)	China	1991–2015	ARDL	Symmetric	The authors find a significant impact of global financial crisis on real and financial sectors in China.
Bai (2019)	China	1990–2017	Granger causality	Symmetric	The author finds significant casual linkages between stock market, human capital, and economic growth.
Peng (2019)	China	1998–2008	DGEM	Symmetric	The author finds that the structural reforms ease business formation and growth led to higher aggregate output based on the resource reallocation resulting from stronger market competition.
Kumar and Paramanik (2020)	India	1996:Q1 to 2018:Q3	NARDL	Asymmetric	The authors find that financial development has a long-run asymmetric impact on economic growth in India.
Vo et al. (2020)	China	1970–2015	ARDL	Symmetric	The authors find a long-run relationship between financial integration and economic growth. They also document a bidirectional causality between the indicators of economic growth and financial integration.
Alenoghena et al. (2020)	Nigeria	1980–2018	NARDL	Asymmetric	The authors find that financial development indicators have a long-run bound with economic growth and they show a U-shaped asymmetric relationship.

Notes: ARDL: Autoregressive distributed lags, VAR: Vector autoregressive, IRF: Impulse response function, PCA: Principal component analysis, DEGM: Dynamic general equilibrium model, NARDL: Non-linear autoregressive distributed lags.

## 3 Methods

### 3.1 Data

The dataset contains quarterly time series ranging from 2003Q1 to 2019Q1 concerning macroeconomic indicators in China from the WDI (World Development Indicators) sources that are relevant to the World Bank Group and available at (https://databank.worldbank.org/). The variables used in the present study are consistent with the theoretical framework in finance and the recent empirical literature (Anderu, 2020; Mishra et al., 2010; Ogbuji et al., 2020; Pokharel, 2020), and they include (i) per capita GDP growth as the dependent variable, (ii) market capitalization as a percentage of the GDP, (iii) total stock traded value to GDP, (iv) stock market turnover, (v) money market rate, (vi) real interest rate, (vii) annual growth rate of the broad money as a proxy for total liquidity, (viii) gross fixed capital formation to GDP as a proxy for capital investment, and (ix) net foreign direct investment inflows to GDP. The indicators (ii-iv) are used to measure the capital market, the indicators (v-vii) are used to measure the money market, and the indicators (viii and ix) are used as the control variables (see: Table 2 for complete variable definition and summary statistics). For better results, time-series econometric analysis encourages the use of high-frequency observations. At the same time, the required dataset for more periods before the year 2003 is not available for the indicators used in the present study. Therefore, this study follows Asogu (1997) and Azimi and Shafiq (2020) for the linear interpolation of the data and converts the original annual dataset into quarterly observations. The quarterly series holds the same property without any difference in the trend and magnitude of the data.

**Table 2:** Variables definitions and summary statistics.

Symbols	Variables' definitions	Mean	Std. Dev.	Min.	Max.	Obs.
**Dependent variable**						
PCGDG	Per capita GDP growth (annual %)	8.565	2.140	5.571	13.635	Qty.
**Money market variables**						
TL	Total liquidity proxy by M3 (broad money annual growth %)	15.341	4.957	6.991	28.423	Qty.
INT	Real interest rate (annual %)	1.636	1.964	-2.305	5.531	Qty.
MMR	Money market rate (%)	2.140	1.971	-2.330	5.450	Qty.
**Capital market variables**						
MC	Market capitalization (GDP %)	54.297	20.937	17.579	126.153	Qty.
ST	Total stock traded value to GDP	112.522	70.548	17.164	355.519	Qty.
SMT	Stock market turnover	206.716	94.313	77.304	556.912	Qty.
**Control variables**						
NFDI	Net FDI inflows to GDP	3.017	1.055	1.091	4.554	Qty.
CIN	Gross fixed capital formation to GDP % as a proxy for capital investment	41.716	2.209	37.892	44.518	Qty.

Notes: Sample size adjusted from 2003Q1 to 2019Q1. All data are collected from WDI official website. FDI: Foreign direct investment, GDP: Gross domestic product, Min.: Minimum, Max.: Maximum, Std. Dev.: Standard Deviation, Obs.: Observations, Qty.: Quarterly observations.

Table 2 describes the variables and provides summary statistics relevant to the indicators used in the present study. It reveals that the dependent variable, per capita GDP growth, shows an average of 8.565% and a maximum of 13.635%, which indicates a comparatively fair increase in per capita GDP growth during the period in China. On the regressors' front, market capitalization (MC) shows a mean value of 54.297% of GDP, indicating a stimulus to economic growth in China. The total stock traded value to GDP (ST) shows an average value of 112.522 to the GDP, which reveals the increase in boosting the capital base of the listed firms (Ogbuji et al., 2020) in China during the period under this study. The average value of the stock market turnover (SMT) stands at 206.716 with a minimum of 77.304 and a maximum value of 556.912 that represents a relatively high stock turnover that stimulates the economic growth in China. The total liquidity (TL) presents an average growth of 15.341%, the real interest rate (INT) stands at an average of 1.636%, and the money market rate (MMR) shows an average of 2.140%. On the control variables, one can see that the average net FDI inflows to GDP (NFDI) is 3.017. It is expected that FDI is the source of funds for acquisitions that spur economic growth (Yousaf et al., 2011). Finally, the summary statistics show that capital investment (CIN) averaged at 41.716 to GDP during the study period. Next, Table 3 presents the results of the correlation coefficients in a matrix format. The results indicate that PCGDPG being the dependent variable is not significantly correlated with the money market, capital market, and control variables.

**Table 3:** Correlation results.

Correlation Matrix	PCGDPG	TL	INT	MMR	MC	ST	SMT	NFDI	CIN
PCGDPG	1								
TL	0.417	1							
INT	0.012	0.034	1						
MMR	0.332	0.868	-0.162	1					
MC	-0.490	-0.401	-0.174	-0.532	1				
ST	-0.655	-0.459	0.081	-0.560	0.923	1			
SMT	0.365	0.514	0.566	0.380	-0.497	-0.445	1		
NFDI	0.251	0.339	0.711	0.182	-0.473	-0.369	0.961	1	
CIN	-0.234	-0.092	-0.017	-0.300	0.713	0.709	-0.206	-0.200	1

Notes: PCGDPG: Per capita GDP growth, TL: Total liquidity, INT: Real interest rate, MMR: Money market rate, MC: Market capitalization, ST: Total stock traded, SMT: Stock market turnover, NFDI: Net foreign direct investment, CIN: Capital investment.

Before estimating the base model to test the hypotheses, it is important to check whether there is a perfect collinearity among the variables. In the existence of perfect collinearity, it is hard to distinguish the individual effects of the explanatory variables on the outcome variable, while their joint effect remains unaffected. Since there is no perfect collinearity among the indicators and the present study employs an asymmetric model, the estimated coefficients of the base model, therefore, the NARDL model, are consistent and unbiased. Following the correlation analysis, it is quite important to understand the explanatory power of the money market and capital market indicators on the dependent variable. Therefore, the PCA (principal component analysis) technique is applied and the results are reported in Table 4.

**Table 4:** Principal component analysis results.

Components	Eigen	Variance %	Cumulative proportion %	First PC	Second PC	Third PC
1	4.3525	48.36	48.36	0.301	-0.145	0.025
2	1.9912	22.12	70.49	0.329	-0.104	0.585
3	1.2953	14.39	84.88	0.134	0.610	-0.109
4	0.7367	8.19	93.06	0.33	-0.278	0.438
5	0.3229	3.59	96.65	-0.418	0.123	0.293
6	0.2092	2.32	98.98	-0.416	0.261	0.231
7	0.0793	0.88	99.86	0.372	0.388	0.127
8	0.0106	0.12	99.98	0.331	0.491	-0.016
9	0.0022	0.02	100.00	-0.28	0.212	0.546

Notes: Sample size adjusted from 2003Q1 to 2019Q1. PCA extraction is based on 9 of 9 indicators loaded in the system. Eigen values: (sum = 9, average = 1). PC: Principal component.

The results of the principal component analysis reveal that components one to nine posit explanatory power ranging from 48% to 12%, which implies that in the first component, 48% of the variation of the indicators is considered matched with less than 12%. Therefore, it notes that the first component allows for the maximum explanatory power to fairly account for the variations in the capital and money market variables, and therefore it only interprets the first component. Moreover, the estimated values in the last-right column are the values that consider the final depth variables that are fed into the subsequent equations.

### 3.2 Econometric methods

Adopting all the estimating procedures used by Chen et al. (2020), this section explains the econometric methods used to examine the asymmetric effects of capital and money market indicators on economic growth, which is the asymmetric ARDL, and therefore, the non-linear ARDL model of Shin et al. (2014). In the present study, employing the asymmetric ARDL model is based on its superiority over the linear ARDL model, as the former allows including the possibility of non-linear effects of positive and negative changes in the independent indicators, whereas the latter does not. Besides, the asymmetric ARDL model provides the estimation and graphical representation of cumulative dynamic multipliers to delve into the adjustment outlines following the positive and negative shocks to the outcome variable (Menegaki, 2019). Moreover, the asymmetric ARDL model allows asymmetric shifts from short-run to long-run effects (see, for instance, Chen et al., 2020). In this faith, the present study initiates the empirical modeling from symmetric ARDL method proposed by Pesaran et al. (2001). Thus, let $y_{t}$ and $x_{t}$ be a set of bivariate macroeconomic indicators showing mixed integrating orders of *I(0)* and *I(1)* without any *I(2)* series. The symmetric ARDL model is expressed as:

(1) $$\Delta y_{t}=\varphi_{y}y_{t-1}+\varphi_{x}x_{t-1}+\lambda k_{t}+\sum_{i=1}^{u-1}\eta_{iy}\Delta y_{t-i}+\sum_{i=1}^{v-1}\eta_{ix}\Delta x_{t-i}+\varepsilon_{t}$$

where the change sign Δ, *φ, η, λ*, and *ε* respectively present the long-run coefficients, short-run coefficients, vector of deterministic regressors considered as exogenous like trend, and the error term. First, it is to test for cointegration. Therefore, Eq. (1) is cointegrated if $y_{t}$ and$x_{t}$ have a long-run nexus and reject the null hypothesis of no cointegration either $\varphi_{y}=\varphi_{x}=0$ jointly or $\varphi_{y}=0,\;\varphi_{x}=0$ separately using the Wald test (Chen et al., 2020) that respectively results in F-statistics and t-statistics. If the F-statistics are greater than the critical value, say, at 5% for the upper bound, the cointegration is confirmed, and if the F-statistics are less than the critical value, say, at 5% for the lower bound, the null hypothesis of no cointegration cannot be rejected. One can note that if the calculated F-statistics fall between the critical values of the lower and upper bounds, say, at 5%, the test is inconclusive about the null hypothesis. Considering the econometric literature (see, for instance, Pesaran et al., 2001; Shin et al., 2014), the ARDL model has several comparative advantages over the cointegrating regressions. The present study does not aim to discuss the advantages; rather, it aims to explore the asymmetric foundation of the model. This is because if $y_{t}$ and$x_{t}$ have non-linear relationships, the use of linear models and symmetric assumptions produces inconsistent results (Anderson et al., 2014), leading to delusive policy inferences. To this faith, the asymmetric ARDL model proposed by Shin et al. (2014) captures the stated empirical shortcomings. The asymmetric ARDL approach decomposes the positive and negative partial sum of squares to implore the asymmetric effects of both long and short runs of $x_{t}$ on$y_{t}$ and produces consistent and spurious-free results, assuming non-linearity. Considering the present study, let$eg_{t}$, $cm_{t}$, and $mm_{t}$ respectively represent economic growth, the capital market, and the money market. Thus, Eq. (1) can be modified into the following long-run linear asymmetric equation:

(2) $$eg_{t}=\varphi^{+}cm_{t}^{+}+\varphi^{-}cm_{t}^{-}+\varphi^{+}mm_{t}^{+}+\varphi^{-}mm_{t}^{-}+u_{t}$$

where $\varphi^{+}\left(\varphi^{-}\right)$ are the positive (negative) partial sums of changes in$cm_{t}$ and $mm_{t}$ that are incorporated by the function $cm_{t}=cm_{t}+cm_{t}^{+}+cm_{t}^{-}$ and$mm_{t}=mm_{t}+mm_{t}^{+}+mm_{t}^{-}$ based on the following process:

(3) $$\begin{array}{l} cm_{t}^{+}=\sum_{j=1}^{t}\Delta cm_{j}^{+}=\sum_{j=1}^{t}\max\left(\Delta cm_{j},0\right),\;cm_{t}^{-}=\sum_{j=1}^{t}\Delta cm_{j}^{-}=\sum_{j=1}^{t}\min\;\left(\Delta cm_{j},0\right),\\ mm_{t}^{+}=\sum_{j=1}^{t}\Delta mm_{j}^{+}=\sum_{j=1}^{t}\max\left(\Delta mm_{j},0\right),\;mm_{t}^{-}=\sum_{j=1}^{t}\Delta mm_{j}^{-}=\sum_{j=1}^{t}\min\;\left(\Delta mm_{j},0\right) \end{array}$$

The linear *I(0)* combination $\left(\psi_{t}\right)$ of Eq. (2) and non-linear partial squares would be:

(4) $$\psi_{t}=k+\lambda_{1}^{+}eg_{t}^{+}+\lambda_{2}^{-}eg_{t}^{-}+\varphi_{1}^{+}cm_{t}^{+}+\varphi_{2}^{-}cm_{t}^{-}+\varphi_{3}^{+}mm_{t}^{+}+\varphi_{4}^{-}mm_{t}^{-}+u_{t}$$

In Eq. (4), stationarity is achieved if $\psi_{t}=I\left(0\right)$ and with the linear long-run asymmetric cointegration for rejected $H_{0}:\lambda_{1}^{+}=\lambda_{2}^{-}=\varphi_{1}^{+}+\varphi_{2}^{-}=\varphi_{3}^{+}+\varphi_{4}^{-}=0$. Although Eqs. (2) and (4) postulate to have multicollinearity and endogeneity problems that need to be adjusted before analyzing the cointegration, the dynamic form of Eqs. (2) and (4) are rationale to identify them. To this faith, Eqs. (2) and (4) can be reconsidered as follows:

(5) $$eg_{t}=\sum_{i=1}^{p}\xi un_{t-i}+\sum_{i=0}^{q}\left(\varphi_{1}^{+}cm_{t}+\varphi_{2}^{-}cm_{t}+\varphi_{3}^{+}mm_{t}+\varphi_{4}^{-}mm_{t}\right)+u_{t}$$

where ξ presents the autoregressive parameter andφ(1−4) are used as the dynamic adjusting parameters including cointegrating dynamism. This takes the following form:

(6) $$\begin{array}{l} \Delta y_{t}=\rho y_{t-1}+\lambda_{1}^{+}cm_{t-1}^{+}+\lambda_{2}^{-}cm_{t-1}^{-}+\lambda_{3}^{+}mm_{t-1}^{+}+\lambda_{4}^{-}mm_{t-1}^{-}\\ \;+\sum_{i=1}^{p}\delta_{i}\Delta y_{t-i}+\sum_{i=1}^{q}\vartheta_{i,1}^{+}\Delta cm_{t-i}^{+}+\vartheta_{i,2}^{-}\Delta cm_{t-i}^{-}\\ \;+\vartheta_{i,3}^{+}\Delta mm_{t-i}^{+}+\vartheta_{i,4}^{-}\Delta mm_{t-i}^{-}+e_{t} \end{array}$$

Eq. (6) is asymmetric, say, non-linear ARDL model proposed by Shin et al. (2014), in which $\varpi^{+}=-\lambda^{+}/\rho$ and$\varpi^{-}=-\lambda^{-}/\rho$, δ andϑ, and $e_{t}$ are respectively the long-run asymmetries, short-run dynamics, and the stochastic error term. One can assume that Eq. (6) is cointegrated if based on F-statistics the null hypothesis of no cointegration is rejected (Pesaran et al., 2001). Since foreign direct investments and the capital investment indicators also affect economic growth, controlling for these variables results to produce better estimates of the long (short) run asymmetries. Therefore, Eq. (6) is modified using the control variables (π) as follows:

(7) $$\begin{array}{l} \Delta y_{t}=\rho y_{t-1}+\lambda_{1}^{+}cm_{t-1}^{+}+\lambda_{2}^{-}cm_{t-1}^{-}+\lambda_{3}^{+}mm_{t-1}^{+}+\lambda_{4}^{-}mm_{t-1}^{-}+\lambda_{5}^{+}\pi_{t-1}^{+}+\lambda_{6}^{-}\pi_{t-1}^{-}\\ \;+\sum_{i=1}^{p}\delta_{i}\Delta y_{t-i}+\sum_{i=1}^{q}\vartheta_{i,1}^{+}\Delta cm_{t-i}^{+}+\sum_{i=1}^{q}\vartheta_{i,2}^{-}\Delta cm_{t-i}^{-}+\sum_{i=1}^{q}\vartheta_{i,3}^{+}\Delta mm_{t-i}^{+}\\ \;+\sum_{i=1}^{q}\vartheta_{i,4}^{-}\Delta mm_{t-i}^{-}\;+\sum_{i=1}^{q}\vartheta_{i,5}^{-}\pi_{t-i}^{-}+\sum_{i=1}^{q}\vartheta_{i,6}^{-}\pi_{t-i}^{-}+e_{t} \end{array}$$

Moreover, the present study investigates how the $\left(eg_{t}\right)$ responds in the long run to standard asymmetric shocks in the capital market and money market using the dynamic multiplier. The dynamic multiplier is used to expedite the sequential growth element as it changes from the milieus of the previous short-run dynamism and the early non-stabilities into a new equilibrium after a standard shock. The equation used is expressed as follows:

(8) $$\begin{array}{l} mh^{+}=\sum_{i=0}^{h}\frac{\partial \left(eg_{t}\right)}{\partial \left(cm_{t}^{+}\right)}=\sum_{i=0}^{h}\varphi_{i}^{+},\;mh^{-}=\sum_{i=0}^{h}\frac{\partial \left(eg_{t}\right)}{\partial \left(cm_{t}^{-}\right)}=\sum_{i=0}^{h}\varphi_{i}^{-},\\ mh^{+}=\sum_{i=0}^{h}\frac{\partial \left(eg_{t}\right)}{\partial \left(mm_{t}^{+}\right)}=\sum_{i=0}^{h}\varphi_{i}^{+},\;mh^{-}=\sum_{i=0}^{h}\frac{\partial \left(eg_{t}\right)}{\partial \left(mm_{t}^{-}\right)}=\sum_{i=0}^{h}\varphi_{i}^{-} \end{array}$$

where $mh^{+}\left(mh^{-}\right)$ are the asymmetric long-run coefficients that are empirically consistent whenm tends to infinity. As a robustness check, granger causality test is performed to test any potential endogeneity issue. Finally, the present study employs several post-estimation tests to ensure consistent and spurious-free results. These tests are: Breusch-Pegan Godfrey, Breusch Godfrey, Jarque-Berra, Remesy, CUSUM (cumulative sum), and the CUSUMSQ (cumulative sum of squares) in allowing for important post-estimate examinations such as the heteroscedasticity, serial correlation, normal distribution of the residuals, and model stability.

## 4 Results and discussions

This section begins with the unit root analysis to test $H_{0}:\delta =0$ (non-stationary series) against $H_{0}:\delta \neq 0$ (stationary series) using the augmented Dickey and Fuller (1979), the Phillips and Perron (1988), and the Ng and Perron (2001) tests to trace out and exclude any indicator sowing I(2) series from the asymmetric ARDL model (Shrestha and Bhatta, 2018). The results of the unit root tests are reported in Table 5 and show that the indicators follow mixed integrating orders. The dependent variable PCGDPG (per capita GDP growth) is an *I(1)* series as it becomes stationary after the first difference.

**Table 5:** Unit root test results.

Variables	Augmented Dickey-Fuller		Phillips-Perron		Ng & Perron	
	Constant	Constant and trend	Constant	Constant and trend	Constant	Constant and trend
**At level**						
PCGDPG	-1.390 [0.518]	-3.855∗∗ [0.019]	-0.740 [0.828]	-2.541 [0.307]	-2.431	-5.953
MMR	-1.756 [0.398]	-2.847 [0.187]	-2.283 [0.180]	-2.622 [0.272]	-7.494	-7.759
INT	-1.895 [0.332]	-2.354 [0.398]	-2.551 [0.108]	-2.859 [0.182]	-7.953	-7.612
TL	-0.632 [0.854]	-2.177 [0.492]	-1.206 [0.666]	-2.105 [0.533]	-4.379	-7.208
MC	-3.914∗∗∗ [0.008]	-3.889∗∗∗ [0.004]	-3.499∗∗∗ [0.009]	-3.520∗∗∗ [0.007]	-19.863∗∗∗	-18.254∗∗
SMT	-2.652∗ [0.088]	-3.223∗ [0.089]	-2.270 [0.184]	-2.413 [0.369]	-6.878	-7.910
ST	-3.812∗∗∗ [0.009]	-3.828∗∗∗ [0.002]	-3.860∗∗∗ [0.002]	-3.705∗∗∗ [0.005]	-14.021∗∗∗	-23.945∗∗∗
NFDI	-0.842 [0.799]	-3.961∗∗ [0.015]	-0.203 [0.932]	-2.620 [0.273]	-1.672	-5.990
CIN	-2.051 [0.246]	-2.407 [0.372]	-1.662 [0.445]	-1.509 [0.816]	-5.077	-10.581
**At first difference**						
PCGDPG	-3.053∗∗ [0.035]	-3.083∗∗ [0.024]	-3.276∗∗ [0.020]	-3.313∗ [0.073]	-16.779∗∗∗	-18.467∗∗
MMR	-3.322∗∗∗ [0.008]	-2.354 [0.398]	-3.867∗∗∗ [0.003]	-3.869∗∗ [0.019]	-23.597∗∗∗	-23.038∗∗∗
INT	-3.488∗∗∗ [0.009]	-3.468∗∗ [0.012]	-3.737∗∗∗ [0.005]	-3.703∗∗ [0.029]	-19.697∗∗∗	-18.277∗∗
TL	-3.173∗∗∗ [0.009]	-3.161∗∗∗ [0.012]	-3.729∗∗∗ [0.005]	-3.720∗∗ [0.028]	-17.347∗∗∗	-18.564∗∗
MC	-4.003∗∗∗ [0.000]	-4.093∗∗∗ [0.001]	-3.872∗∗∗ [0.000]	-3.852∗∗∗ [0.008]	-11.810∗∗∗	-12.177
SMT	-3.878∗∗∗ [0.000]	-2.826 [0.194]	-3.901∗∗∗ [0.003]	3-.891∗∗∗ [0.009]	-19.161∗∗	-23.064∗∗∗
ST	-3.652∗∗∗ [0.002]	-3.706∗∗∗ [0.001]	-3.796∗∗∗ [0.000]	-3.781∗∗∗ [0.001]	-14.091∗∗∗	-25.601∗∗∗
NFDI	-3.218∗∗ [0.023]	-3.338∗ [0.069]	-3.467∗∗ [0.012]	-3.592∗∗ [0.038]	-11.357∗∗	-24.244∗∗∗
CIN	-3.587∗∗∗ [0.001]	-3.579∗∗∗ [0.003]	-3.740∗∗∗ [0.004]	-3.743∗∗∗ [0.009]	-19.917∗∗∗	-19.042∗∗

Notes: ∗∗∗,∗∗,∗ present significance at 1%, 5%, and 10%, respectively. ^a^ Lag length is selected using SIC (Schwarz Information Criterion). Sample size adjusted from 2003Q1 to 2019Q1. PCGDPG: Per capita GDP growth, INT: Real interest rate, TL: Total liquidity, MC: Market capitalization, SMT: Stock market turnover, ST: Total stock traded, NFDI: Net foreign direct investments, CIN: Capital investments. Critical values for Ng. Perron test come from the Ng and Perron (2001) table.

The money market variables, namely, MMR (money market rate), INT (real interest rate), and TL (total liquidity) are also *I(1)* series as their corresponding p-values for the ADF (augmented Dickey-Fuller) and the PP (Philips-Perron) tests are greater than 0.05 at the level, while their test statistics are lower than the critical values at 5% for the Ng and Perron test. Thus, one cannot reject the null hypothesis of non-stationarity. The money market variables become stationary after the first difference as their corresponding p-values are less than 0.01 and 0.05. Therefore, they are *I(1)* series. On the capital market variables’ front, the MC (market capitalization) and the ST (total stock traded) are *I(0)* series displaying the corresponding p-values both for the ADF and the PP tests that are less than 0.01 and 0.05 supported by smaller test statistics of the Ng and Perron than the critical values at 5%, while INT (real interest rate) is non-stationary at the level but it becomes stationary after the first difference. Lastly, the control variables, namely, the NFDI (net foreign direct investments) and the CIN (capital investments) are integrated of first order, which means that they become stationary after the first difference.

Since the variables, namely, economic growth, money market, capital market, and the control variables follow mixed integrating orders, say, *I(0)* and *I(1)* without any *I(2)* series, the study proceeds to estimate and analyze the asymmetric ARDL model. However, the results of the Pesaran et al. (2001) ARDL bound test to cointegration based on Eq. (7) are also reported in Table 6. The estimation of Eq. (7) is based on automatically selected lags using the Akaike information criterion, the Schwarz criterion, and the Hannan-Quinn criterion. The results of bound testing indicate that (F = 4.122; critical value for *I(1) = 3.39*) and (*t= -4.807*; critical value for *I(1) = -4.72*) are significant at 5% to reject the null hypothesis of no cointegration in favor of its alternative hypothesis, implying that there is a long-run asymmetric relationship among the indicators. Moreover, Table 7 reports the results of the Wald test used to test the null $H_{0}:\lambda_{1}^{+}=\lambda_{2}^{-}=\varphi_{1}^{+}+\varphi_{2}^{-}=\varphi_{3}^{+}+\varphi_{4}^{-}=0$ against its alternative $H_{A}:\lambda_{1}^{+}\neq \lambda_{2}^{-}\neq \varphi_{1}^{+}+\varphi_{2}^{-}\neq \varphi_{3}^{+}+\varphi_{4}^{-}\neq 0$. The results indicate that the corresponding p-values of both short and long runs statistics are (W_SR_ = 0.000 < 0.01, W_LR_ = 0.000 < 0.01) supporting the rejection of null hypothesis in favor of its alternative. This implies that in the short and long run, the positive and negative partial sum of square effects and levels of capital and money market variables on economic growth are different. In sum, the results of the Wald test provide significant evidence to reject the null and support the asymmetric behavior of the indicators.

**Table 6:** Bound testing results.

Statistics		Significance	Critical values	
			I(0)	I(1)
F-statistics	4.122∗∗∗	5%	2.22	3.39
t-statistics	-4.807∗∗∗	5%	-2.86	-4.72

Notes: ∗∗∗,∗∗,∗ present significance at 1%, 5%, and 10%, respectively. Sample size spans from 2003Q1 to 2019Q1 after lags adjustment selected by AIC, SC, and HQ criterions. Critical values for lower bound I(0) and upper bound I(1) are collected from Pesaran et al. (2001).

**Table 7:** Symmetric test results.

Wald statistics	Test statistics [p-value]
W_SR_, $\sum_{j=0}^{p-1}n_{j}^{*}=\sum_{j=0}^{p-1}n_{j}^{-}$	264824.4∗∗∗ [0.000]
W_LR_, $-\theta^{*}/\varphi =-\theta^{-}/\varphi$	7679908.∗∗∗ [0.000]

Notes: ∗∗∗, ∗∗, ∗ present significance level at 1%, 5%, and 10%, respectively. Sample size spans from 2003Q1 to 2019Q1 after lags adjustment. The null hypothesis $H_{0}:\lambda_{1}^{+}=\lambda_{2}^{-}=\varphi_{1}^{+}+\varphi_{2}^{-}=\varphi_{3}^{+}+\varphi_{4}^{-}=0$ (symmetries) against $H_{A}:\lambda_{1}^{+}\neq \lambda_{2}^{-}\neq \varphi_{1}^{+}+\varphi_{2}^{-}\neq \varphi_{3}^{+}+\varphi_{4}^{-}\neq 0$ (asymmetries). W_SR_ and W_LR_ respectively are the short-run and the long-run asymmetries.

Since the results obtained from the Wald test reveal that capital and money market indicators differently affect economic growth at different levels both in the short and long runs, it triggers further statistical analysis to explore their level of positive and negative impacts on economic growth (Chen et al., 2020). To this faith, the present study computes the asymmetric ARDL model and reports its results in Table 8. These results show, on the one hand, that $MMR_{1,t-1}^{+}$ (positive partial sum of squares of money market rate) has an asymmetrically significant impact on economic growth, implying that positive shocks from the money market rate spur economic growth by 0.221%, while on the other, $MMR_{1,t-1}^{-}\;$ shows a significant coefficient of -0.228, meaning that the negative shocks from the money market rate decrease economic growth by 0.228%. The $INT_{1,t-1}^{+}$ (positive partial sum of squares of the real interest rate) shows a significant coefficient of -0.154, implying that a unit increase in the real interest rate decreases economic growth by 0.154%, while its negative partial sum demonstrates that with a unit decrease in the real interest rate, economic growth increases by 0.162%. This result is related to the fact that low interest rates encourage borrowers to borrow more money to finance business expansion, but an increase in the cost of money, say, the interest rate, means less cash is available to finance business needs. Therefore, in aggregate, a decrease in interest rates increases economic growth, while its increase has reverted behavior in the economy.

**Table 8:** Asymmetric results of the ARDL estimates.

Dependent variable: Per capita GDP growth		Asymmetric ARDL models estimates					
		$MMR_{t-1}^{+}$	$MMR_{t-1}^{-}$	$INT_{t-1}^{+}$	$INT_{t-1}^{-}$	$TL_{t-1}^{+}$	$TL_{t-1}^{-}$
Money market variables							
Coefficients		0.211∗∗∗	-0.228∗∗∗	-0.154∗∗∗	0.162∗∗∗	-0.193∗∗∗	0.138∗∗∗
Std. Error		0.036	0.037	0.052	0.055	0.011	0.026
t-statistics		5.873	-6.110	-2.972	2.960	-18.276	5.236
Capital market variables		$MC_{t-1}^{+}$	$MC_{t-1}^{-}$	$SMT_{t-1}^{+}$	$SMT_{t-1}^{-}$	$ST_{t-1}^{+}$	$ST_{t-1}^{-}$
Coefficients		-0.035∗∗∗	-0.041∗∗∗	-0.009∗∗∗	-0.019∗∗∗	0.014∗∗∗	0.036∗∗∗
Std. Error		0.004	0.002	0.002	0.002	0.003	0.003
t-statistics		-8.724	-17.614	-3.770	-11.901	4.554	13.578
Control variables		$NFDI_{t-1}$	$CIN_{t-1}$				
Std. Error		0.070	0.051				
t-statistics		-20.846	4.008				
Coefficients		$PCGDPG_{t-1}$					
Std. Error		0.631∗∗∗					
t-statistics		0.082					
		7.695					
Diagnostic tests	Ad.R^2^	BPG	BG	JB	Remesy	CUSUM	CUSUMSQ
0.999	21.436	0.434	0.818	0.331	Stable	Stable	
	[0.645]	[0.514]	[0.764]	[0.551]			

Notes: ∗∗∗,∗∗,∗ present significance at 1%, 5%, and 10%, respectively. (+) positive partial sum, (-) negative partial sum. Sample size adjusted from 2003Q1 to 2019Q1. AIC: Akaike information criterion, INT: Real interest rate, TL: Total liquidity, MC: Market capitalization, SMT: Stock market turnover, ST: Total stock traded, NFDI: Net foreign direct investments, CIN: Capital investments, BPG: Bresch Pegan Godfrey, BP: Bruesch Pegan, JB: Jarque-Berra.

With total liquidity, the positive partial sum $TL_{1,t-1}^{+}$ and the negative partial sum $TL_{1,t-1}^{-}$ respectively, imply that positive shocks from total liquidity decrease economic growth by 0.193%, while its negative shocks spur economic growth by 0.138%. Liquidity is an integral component of financial markets and is of interest to investors, businesses, and regulators. Consistent with recent studies (see for instance: Hameed et al., 2010) showing that more liquidity negatively reacts to trading endeavors. The results shown in Table 8 indicate that an increase in total liquidity suppresses economic growth, while a decrease in total liquidity spurs economic growth in China. For brevity, the results indicate that controlling for net foreign direct investments and capital investments, all money market and capital market variables, namely, money market rate, real interest rate, total liquidity, market capitalization, stock market turnover, and the total stock traded, have both positive partial sum and negative partial sum significant impacts on economic growth in China. In exporting the effects of financial development on economic growth, comparatively, Jalil et al. (2010) used the symmetric ARDL and bound testing approach, in which they only provided evidence of a long-run relationship between financial development and economic growth in China, though the magnitude of effect levels were implicitly presented. Under the assumption of symmetrical effects of financial integration on economic growth, Vo et al. (2020) discovered that financial integration symmetrically affected economic growth by -0.844% and -0.766% in the long-run, while the short-run effects were symmetrically positive to influence economic growth in China. Ignoring both control variables and the asymmetrical nexus of finance-growth, their results seem to be confounded by high level effects and reverse magnitude.

On the statistical validation front, all the results obtained and reported in Table 8 are spurious-free. The adjusted r-squared value is 99.9%, showing high goodness of fit and accounting for 99.9% of the variations in the observations. It is important to note that the r-squared value is used to measure the explanation of the dataset variance, rather than the predictive capacity of the NARDL model. The p-values for BPG, BG, JB, and Remesy are 0.645, 0.434, 0.818, and 0.331 respectively, greater than 5% significance, indicating that the estimated results do not suffer from heteroscedasticity, serial correlation, and abnormal distribution of the residuals.

Table 9 reports the short-run and long-run asymmetric effects of capital and money market variables on economic growth using the asymmetric ARDL model. For the short run asymmetries of money market indicators, the positive partial sum of squares of the money market rate decreases economic growth by 0.215%, while its negative shocks increase economic growth by 0.232%. Unlike the assumptions, the positive partial sum of real interest rates spurs economic growth by 0.154%, while its negative partial sum decreases economic growth by 0.163% in the short-run. With total liquidity, the positive partial sum shows that an increase in total liquidity causes economic growth to increase by 0.192%, while its negative partial sum suppresses economic growth by 0.141%. According to Levine (2003) there is a strong nexus between stock market liquidity and economic growth, as higher stock market liquidity improves economic growth both in the short and long runs. Since long-run asymmetries of money market indicators are insignificant, the present study ignores their interpretations.

**Table 9:** Shor-run and long-run asymmetries.

Dependent variable: Per capita GDP growth	Short-run asymmetries: AIC		Long-run asymmetries: AIC		
	Coefficients	t-statistics		Coefficients	t-statistics
$PCGDPG_{t-1}$				0.631∗∗∗	7.695
**Money market variables**					
$\text{Δ}{\text{MMR}}_{\text{t}-1}^{+}$	-0.215	-5.955∗∗∗	${\text{MMR}}_{\text{t}-1}^{+}$	-0.073	-0.661
$\text{Δ}{\text{MMR}}_{\text{t}-1}^{-}$	0.232	6.220∗∗∗	${\text{MMR}}_{\text{t}-1}^{-}$	0.079	0.771
$\text{Δ}{\text{INT}}_{\text{t}-1}^{+}$	0.154	2.946∗∗∗	${\text{INT}}_{\text{t}-1}^{+}$	0.002	0.024
$\text{Δ}{\text{INT}}_{\text{t}-1}^{-}$	-0.163	-2.938∗∗∗	${\text{INT}}_{\text{t}-1}^{-}$	-0.010	-0.044
$\text{Δ}{\text{TL}}_{\text{t}-1}^{+}$	0.192	17.709∗∗∗	${\text{TL}}_{\text{t}-1}^{+}$	-0.011	-0.101
$\text{Δ}{\text{TL}}_{\text{t}-1}^{-}$	-0.141	-5.340∗∗∗	${\text{TL}}_{\text{t}-1}^{-}$	-0.042	-0.310
**Capital market variables**					
$\text{Δ}{\text{MC}}_{\text{t}-1}^{+}$	0.037	9.702∗∗∗	${\text{MC}}_{\text{t}-1}^{+}$	0.039	3.892∗∗∗
$\text{Δ}{\text{MC}}_{\text{t}-1}^{-}$	0.044	22.859∗∗∗	${\text{MC}}_{\text{t}-1}^{-}$	0.051	10.263∗∗∗
$\text{Δ}{\text{SMT}}_{\text{t}-1}^{+}$	0.009	4.161∗∗∗	${\text{SMT}}_{\text{t}-1}^{+}$	0.015	1.671
$\text{Δ}{\text{SMT}}_{\text{t}-1}^{-}$	0.019	11.555∗∗∗	${\text{SMT}}_{\text{t}-1}^{-}$	0.010	2.398∗∗
$\text{Δ}{\text{ST}}_{\text{t}-1}^{+}$	-0.016	-4.991∗∗∗	${\text{ST}}_{\text{t}-1}^{+}$	-0.021	-1.89∗
$\text{Δ}{\text{ST}}_{\text{t}-1}^{-}$	-0.037	-13.214∗∗∗	${\text{ST}}_{\text{t}-1}^{-}$	-0.023	-3.307∗∗∗
**Control variables**					
			NFDI_t-1_	1.279	7.422∗∗∗
			CIN _t-1_	-0.110	-0.817
Diagnostic tests Ad. R2	ECT	BPG	BG	JB	Remesy
0.999	-0.056∗∗∗	23.643	0.843	0.189	0.109

Notes: ∗∗∗,∗∗,∗ present significance at 1%, 5%, and 10%, respectively. (+) positive partial sum, (-) negative partial sum. Sample size adjusted from 2003Q1 to 2019Q1. AIC: Akaike information criterion, INT: Real interest rate, TL: Total liquidity, MC: Market capitalization, SMT: Stock market turnover, ST: Total stock traded, NFDI: Net foreign direct investments, CIN: Capital investments, ECT: Error-correcting model, BPG: Bresch Pegan Godfrey, BP: Bruesch Pegan, JB: Jarque-Berra.

In terms of capital market indicators, for short-run asymmetries, market capitalization shows significant asymmetric effects. It indicates that both positive and negative partial sum of squares of market capitalization increase economic growth by 0.037% and 0.044% respectively, while for the long-run asymmetries, the results indicate that positive and negative partial sums of squares of market capitalization increase economic growth by 0.039% and 0.051% respectively. This is consistent with recent empirical works (see, for instance, Dökmen et al., 2015), which discovered that market capitalization has a significantly positive impact on economic growth. This triggers that countries with higher market capitalization are linked with faster economic growth and output. With the stock market turnover, the short-run asymmetric effects are significant. The results reveal that by positive and negative partial sums, economic growth increases by 0.009% and 0.019%, respectively. Lastly, the total stock traded exhibits significance both for short and long runs. The results show that positive and negative partial sums decrease economic growth by 0.016% and 0.037% in the short-run and by 0.021% and 0.023% in the long-run. For brevity, except for capital market variables that are significantly asymmetric in the long run, all money market indicators are only significantly asymmetric in the short run. Since the asymmetric ARDL model used in the present study is based on the conditional error-correcting method, its results are also computed and reported in the rear part of Table 9. Besides the significant coefficient of the ECT (error-correcting term) that reconfirms the cointegration among the series, it also shows that the short-run asymmetries are restored to their long-run equilibrium by an adjusting speed of 0.056 per quarter. All the results obtained and reported in Table 9 are spurious-free and statistically validated. As an instance, the adjusted r-squared is 0.999, meaning that independent variables explain the dependent variable by 99.9%, which is an indication of a high goodness of fit. The corresponding p-values for BPG, BG, and JB are greater than 5% respectively, indicating that the model does not suffer from heteroscedasticity, serial correlation, and abnormal residual distribution. Next, the variance decomposition of the dependent variable is analyzed to trace out the proportion of the dependent variable explained by money and capital markets predictors. The results are reported in Table 10.

**Table 10:** Variance decomposition results.

Period: Quarterly	S.E.	PCGDPG	MMR	INT	TL	MC	SMT	ST	NFDI	CIN
1	0.200	100.000	0.000	0.000	0.000	0.000	0.000	0.000	0.000	0.000
2	0.335	98.494	0.664	0.301	0.079	0.020	0.033	0.078	0.117	0.211
3	0.423	93.721	0.736	1.373	2.071	0.648	0.148	0.511	0.122	0.666
4	0.495	81.940	0.659	3.028	8.779	3.194	0.400	0.953	0.100	0.942
5	0.579	65.642	1.731	4.406	17.534	8.035	0.789	0.814	0.198	0.847
6	0.671	51.197	3.047	4.999	23.305	14.467	1.278	0.716	0.343	0.644
7	0.760	40.533	3.624	4.803	24.910	21.858	1.844	1.443	0.476	0.504
8	0.845	32.887	3.572	4.070	23.597	29.402	2.463	2.952	0.630	0.422
9	0.927	27.356	3.283	3.434	20.928	35.969	3.083	4.705	0.869	0.368
10	1.008	23.250	2.972	3.692	18.041	40.637	3.626	6.192	1.256	0.330

Notes: Sample size adjusted from 2003Q1 to 2019Q1. PCGDPG: Per capita GDP growth, INT: Real interest rate, TL: Total liquidity, MC: Market capitalization, SMT: Stock market turnover, ST: Total stock traded, NFDI: Net foreign direct investments, CIN: Capital investments, SE: Standard error.

**Figure 1:**
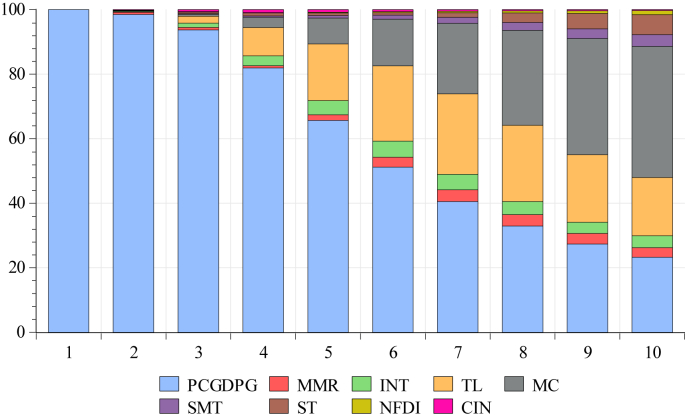
Variance decomposition of PCGDPG.

Table 10 presents the variance decomposition of the dependent variable PCGDPG. The results show that in the short-run, say, in the first quarter, the dependent variable explains itself by 100% while in the following quarters, it reduces to 23.25%. Moreover, the results indicate that from the second quarter onward, the MMR has a relatively fair influence on economic growth in the runs ranging from 0.664% to 2.97%. Among all the other regressors, market capitalization is shown to have a stronger influence on economic growth. From the second quarter onward, it influences the dependent variable by 0.02%, while it exhibits 35.969% in the 9^th^ and 40.637% influence in the 10^th^ quarter. The second most influential regressor is total liability, which is shown to have a strong influence on economic growth. The same results are presented in Figure 1.

**Figure 2:**
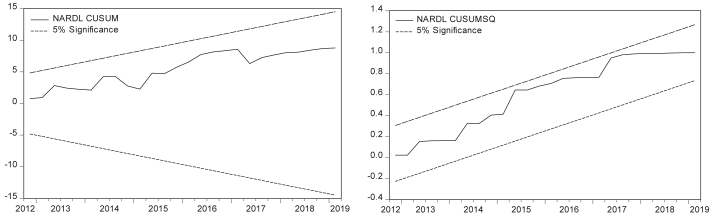
CUSUM and CUSUMSQ test results.

In addition, this study depicts the execution of recursive CUSUM and CUSUMSQ tests against the breakpoints to test the null hypothesis of unstable parameters. The CUSUM and CUSUMSQ results are plotted in Figure 2. At 5% significance, the residuals are shown to be within the bounds and thus confirm the stability of the parameters in the model (see for instance: Brown et al., 1975). Moreover, to test for any potential endogeneity issue, the present study computes the Granger causality test and reports the results in annex A1, Table 11. The results show that there is no causality running from the PCGDPG to any of the explanatory variables. This implies that the statistical results do not suffer from any endogeneity problem.

**Figure 3:**
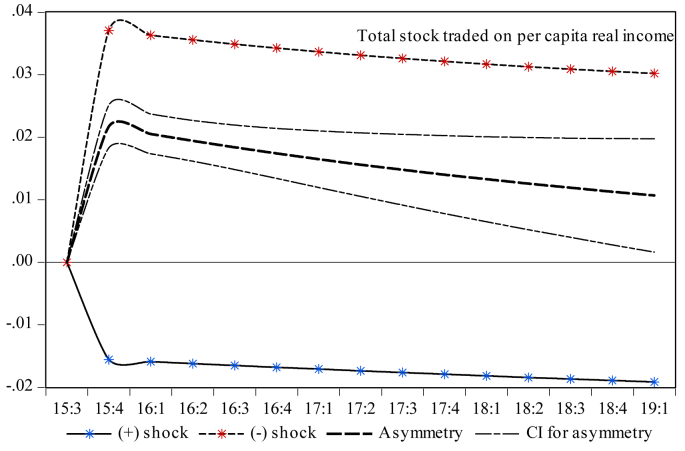
Dynamic multiplier; total stock traded on per capita real income. Note: 95% confidence interval bootstrap is based on 65 replications.

**Figure 4:**
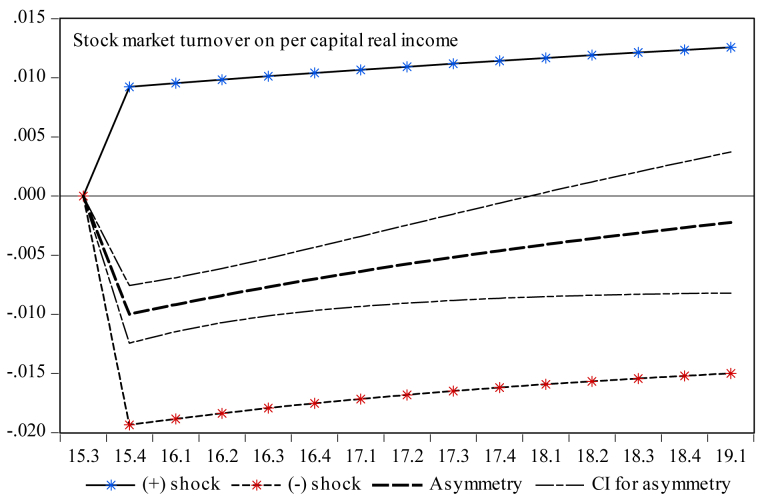
Dynamic multiplier; stock market turnover on per capita real income. Note: 95% confidence interval bootstrap is based on 65 replications.

**Figure 5:**
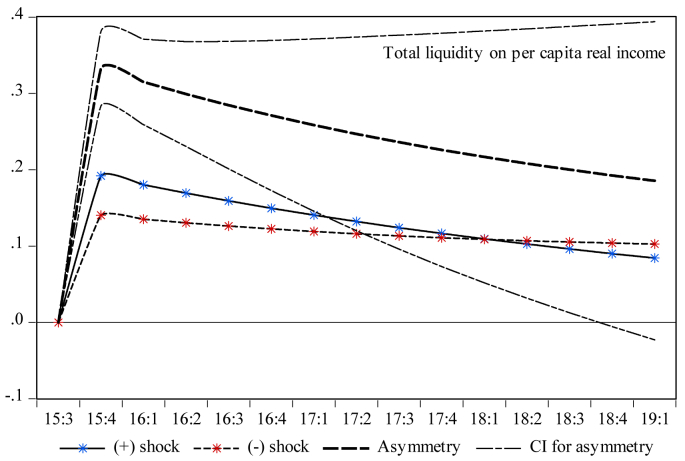
Dynamic multiplier; total liquidity on per capita real income. Note: 95% confidence interval bootstrap is based on 65 replications.

**Figure 6:**
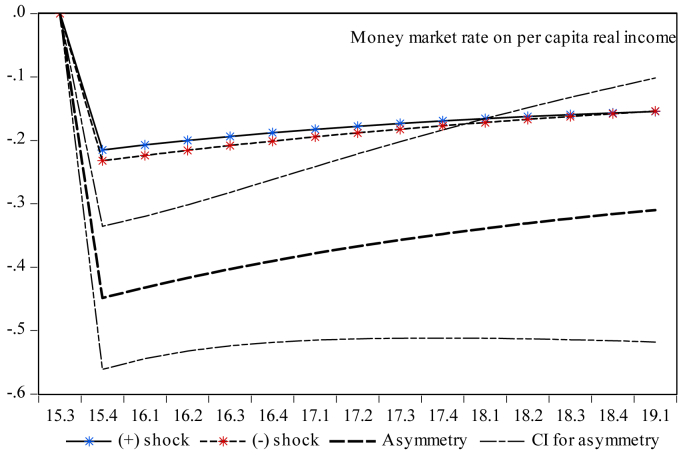
Dynamic multiplier; money market rate on per capita real income. Note: 95% confidence interval bootstrap is based on 65 replications.

**Figure 7:**
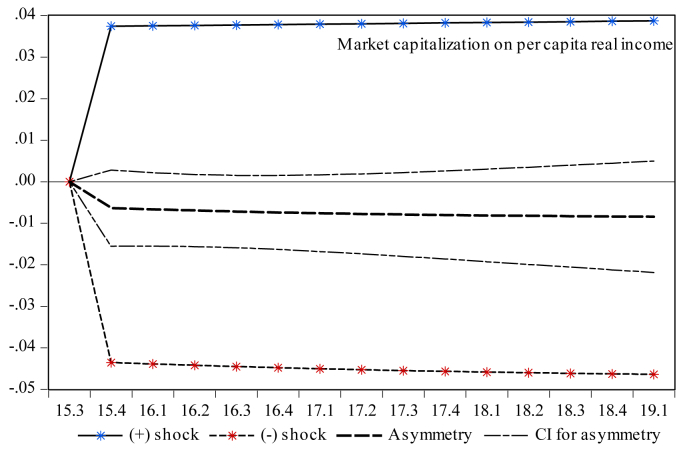
Dynamic multiplier; market capitalization on per capita real income. Note: 95% confidence interval bootstrap is based on 65 replications.

**Figure 8:**
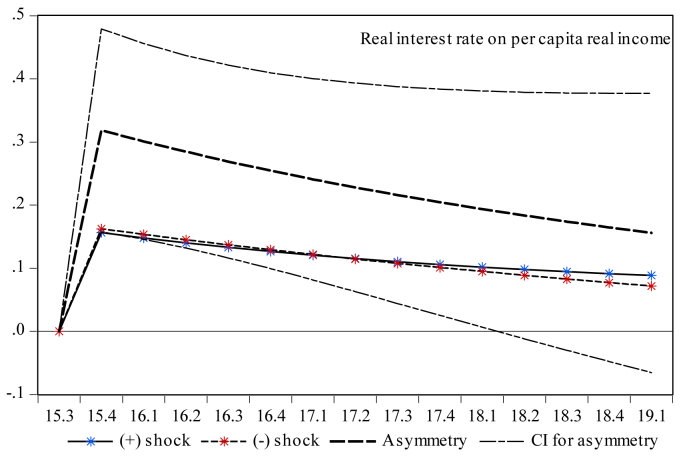
Dynamic multiplier; real interest rate on per capita real income. Note: 95% confidence interval bootstrap is based on 65 replications.

Finally, it is essential to investigate the dynamic multiplier behavior of the temporal dynamics of economic growth to account for the milieus devised by short-run dynamics and the primary disequilibrium due to the shocks to capital and money market indicators. One can note that the rejection of the null hypothesis presented by the results in Table 5 indicates that there is an initial cointegration among the series. Therefore, exploring the dynamic multipliers provides more insights into the statistical validity of the asymmetric results reported in Table 7. Therefore, Figure 3 indicates that a standard deviation shock of both positive and negative partial sums has a reversible impact on economic growth both in the short and long runs. Moreover, Figure 4 shows the counter-behavior of economic growth towards both positive and negative shocks of stock market turnover in spurring economic growth both in the short and long runs. Reading through Figure 5 reveals that a positive standard deviation shock from total liquidity improves economic growth in the short run, whereas economic growth shows diminishing behavior against both positive and negative standard deviation shocks from total liquidity in the long run. One can see in Figure 6 that both negative and positive shocks from the money market rate spur economic growth, while market capitalization exerts a positive asymmetric impact on economic growth both in the short and long runs (see Figure 7). Lastly, Figure 8 presents the results of the dynamic multiplier for the response of economic growth to positive and negative shocks. The results show that economic growth has reverted behavior against both positive and negative shocks from real interest rates.

Comparatively, in the past ten years, market-based financial systems such as the United States and the United Kingdom have performed better than bank-based financial systems such as Japan and Germany, and this has generally been respected as evidence of the market's superiority in the financial system. Although spectators sometimes refer to the "financial revolution" driven by the rise of the US venture capital market, China's financial structure is often seen as not favorable to economic growth. Recent empirical works on this issue have spurred policy initiatives aimed at supporting the market-based financial system in China. According to Shobande and Shodipe (2021), it is important that policymakers formulate national monetary policies to respond to financial and macroeconomic variables that ease financial integration and provide responses to the international orders that release economics shocks through the international financial frameworks.

## 5 Conclusions

This study explores the asymmetric effects of capital and money markets' indicators on economic growth in China using the non-linear ARDL method. The non-linearity approach to testing the short-run and long-run effects is based on the fact that linear assumptions in a non-linear combination of indicators do not provide sufficient and statistically valid evidence to ascertain the effects of finance on economic growth. To test this hypothesis, the present study uses a set of time series data relevant to China's economy for the period from 2003Q1 to 2019Q1 collected from WDI (World Development Indicators). The primary data analysis reveals that the per capita GDP growth, capital market, money market, and control variables are I(0) and I(1) without any I(2) series. Further analysis shows that there is a significant long-run bound amid finance and economic growth. Using the Wald test, the results confirm that capital and money market indicators asymmetrically and differently impact economic growth both in the short and long runs. This study employs the non-linear ARDL model based on a conditional error-correcting mechanism to test the research hypothesis, which is the asymmetric effects of capital and money market indicators on economic growth in China.

The results indicate that except for capital market indicators, namely, market capitalization, stock market turnover, and the total stock traded that exhibit asymmetric long-run effects on economic growth, all other indicators, both for money market and capital market variables are significantly asymmetric in impacting economic growth in the short-run. The most critical results of the present study support the financial supply-led assumptions based on significant results of the short-run asymmetries that can attract sound financial projects leading to sustainable and long-run economic growth. This is also based on the fact that the error-correction results show a -0.056, say, 5.6% quarterly speed of adjustment of the short-run asymmetries to long-run equilibrium.

The critical findings of this study imply important policy implications for China. It is worth emphasizing that financial market development asymmetries are mostly determined by the size and functions of the money and capital markets. Broadly, these are the elements of China's enabling environment, which are more closely linked to capital and money market functions to boost economic growth. Sustained growth, a broad respect for market sovereignty, an effective and equitable legal system, and an efficient and appropriate regulatory environment are all elements that contribute to the development of financial markets and the creation of optimal circumstances for financial transactions. Other factors that are discussed herein are more concerned with maintaining the financial sector's independence and international financial frameworks that transfer shocks to China's economy.

Therefore, on the policy front, based on the critical findings of this study, the following set of policy measures are recommended:

i. Considering the asymmetric impact of financial indicators on economic growth, the findings suggest that controlling for interest rates, sustainable money market rates, maintaining sufficient market liquidity, and supporting stock market trade by adjusting the existing regulations, taxation, and spending programs can further boost economic growth in the long run.
ii. Moreover, policies that support the reduction of interest rates by boosting short-run market activity would be noteworthy for sustainable long-run economic growth.
iii. As the reduction in interest rates spurs growth, effective policies to offset the high liquidity negativities in the market should also be put forward to heighten the financial system's integration.
iv. Unlike the assumptions, the stock market turnover confirms growth suppression. It might be due to limited market liberalization in China and the limitation of permissions for qualified international investors to trade liberally in the market. Boosting booming economic growth implies liberalizing stock market trade and readjusting type A, B, and H share trades in China, or these results could be achieved as data guided.
v. Lastly, equity finance is one of the major factors driving economic growth. Policies that can foster market development should also be put in place in China.

## Declarations

### Author contribution statement

Mohammad Naim Azimi: Conceived and designed the experiments; Performed the experiments; Analyzed and interpreted the data; Contributed reagents, materials, analysis tools or data; Wrote the paper.

### Funding statement

This research did not receive any specific grant from funding agencies in the public, commercial, or not-for-profit sectors.

### Data availability statement

Data associated with this study has been collected from the World Bank, World Development Indicators (WDI) (https://databank.worldbank.org/).

### Declaration of interests statement

The authors declare no conflict of interest.

### Additional information

No additional information is available for this paper.

## Appendix A Supplementary data

Annex Annex
