# Supplementary material for: Assessing the asymmetric effects of capital and money markets on economic growth in China
Source: Heliyon. 2022 Jan 21;8(1):e08794. doi: 10.1016/j.heliyon.2022.e08794 (PMC8790501; doi:10.1016/j.heliyon.2022.e08794)
Supplement: Annex [file mmc1.docx]

Annex A1

**Table 11** Evidence of no endogeneity.

| **Causality directions** | **Significant level** | **F-statistics**  **[p-value]** |  |
| --- | --- | --- | --- |
| **Money market variables** | | |  |
| PCGDPG does not cause MMR | 1% | 1.481 [0.2431] |  |
| PCGDPG does not cause INT | 1% | 0.421 [0.850] |  |
| PCGDPG does not cause TL | 1% | 1.377 [0.258] |  |
| **Capital market variables** | | |  |
| PCGDPG does not cause MC | 1% | 0.630 [0.536] |  |
| PCGDPG does not cause SMT | 1% | 2.044 [0.138] |  |
| PCGDPG does not cause ST | 1% | 1.409 [0.252] |  |
| **Control variables** | | |  |
| PCGDPG does not cause NFDI | 1% | 1.505 [0.230] |  |
| PCGDPG does not cause CIN | 1% | 2.276 [0.185] |  |
| Notes: ***,**,* present significance at 1%, 5%, and 10% respectively. ^a^ Lag length is selected using AIC (Akaike Information Criterion). Sample size adjusted from 2003Q1 to 2019Q1. PCGDPG: Per capita GDP growth, INT: Real interest rate, TL: Total liquidity, MC: Market capitalization, SMT: Stock market turnover, ST: Total stock traded, NFDI: Net foreign direct investments, CIN: Capital investments. | | | |
